# Correlation between serum uric acid-to-creatinine ratio and diabetic retinopathy in type 2 diabetes mellitus patients

**DOI:** 10.3389/fendo.2026.1977560

**Published:** 2026-10-19

**Authors:** Lei Wang, Zhangxiang Zhu, Yu Gong, Guojuan Wang

**Affiliations:** 1 Department of Endocrinology and Metabolism, The First People’s Hospital of Hefei, Hefei, China; 2 Bengbu Medical University, Bengbu, China

**Keywords:** diabetic retinopathy, risk factor, serum uric acid, serum uric acid-to-creatinine ratio (SUA/SCr), type 2 diabetes mellitus

## Abstract

**Background:**

Diabetic retinopathy (DR) is a predominant microvascular complication in patients with type 2 diabetes mellitus (T2DM) and constitutes one of the leading causes of vision loss and blindness among this population. Serum uric acid (SUA) acts as a critical biomarker for microangiopathy; however, its relationship with DR remains incompletely elucidated. To adjust for inter-individual heterogeneity and confounding effects of baseline renal function, serum creatinine was used to normalize uric acid concentrations. Accordingly, the serum uric acid-to-creatinine ratio (SUA/SCr) was selected as the study index, as this metric can better reflect the net metabolic burden of uric acid as well as the status of oxidative stress. The present study aimed to explore the association between SUA/SCr levels and DR risk in patients with T2DM.

**Methods:**

A total of 1091 patients diagnosed with T2DM who visited the Department of Internal Medicine at Hefei First People’s Hospital between 2022 and 2024 were initially recruited. Following screening according to pre-defined inclusion and exclusion criteria, 427 eligible subjects were finally enrolled and stratified into DR and non-DR groups. Spearman rank-order correlation analysis was performed to evaluate correlations between DR status and measured clinical variables. Univariable binary logistic regression was performed to screen potential risk factors. Variables with P< 0.05 in the univariable analysis were entered into the baseline multivariable logistic regression model. *Post-hoc* power calculation was performed for the primary multivariable logistic regression. With an assumed odds ratio of 1.22, α = 0.05, and a case-to-noncase ratio of 1:1.3, the sample size of 427 participants provided adequate statistical power to detect the target effect size.Receiver operating characteristic (ROC) curve analysis was utilized to estimate the predictive value of SUA/SCr for DR in T2DM patients. Furthermore, C-statistics were calculated and compared via DeLong’s test to determine whether adding SUA/SCr could enhance the predictive performance of the baseline DR-risk model. Net reclassification improvement (NRI) and continuous integrated discrimination improvement (IDI) were additionally computed to quantify the incremental predictive value contributed by SUA/SCr.

**Results:**

Baseline-characteristic comparisons showed significant between-group differences in age, disease duration, glycated hemoglobin A1c (HbA1c), albumin (ALB), aspartate transaminase (AST), alanine transaminase (ALT), gamma-glutamyl transferase (γ-GT), total bilirubin (TBIL), triglycerides (TG), SUA, and SUA/SCr (all *P<0.05). Spearman correlation analyses demonstrated that DR status was positively correlated with age, disease duration, HbA1c, SUA, and SUA/SCr (all *P<0.05), whereas negative correlations were observed for C-peptide (C-P), ALB, AST, ALT, γ-GT, and TBIL (all *P<0.05). Univariate and multivariable logistic regression indicated that elevated SUA/SCr was associated with higher DR risk among patients with T2DM. ROC-curve analysis revealed that SUA/SCr exhibited favourable discriminative performance for DR, with an AUC of 0.774 (95% CI: 0.720–0.827, *P<0.05). The optimal cut-off value was 3.68, corresponding to a sensitivity of 79.8% and specificity of 67.2%. When SUA/SCr was incorporated into the baseline DR-prediction model, incremental predictive improvement was observed in terms of IDI but not NRI: NRI was 0.140 (95% CI: -0.068–0.348, *P= 0.187), and IDI was 0.021 (95% CI: 0.006–0.036, *P= 0.006).

**Conclusion:**

SUA/SCr was significantly associated with DR in patients with T2DM. Although its incremental predictive improvement was modest, SUA/SCr yielded significant additional discriminative power for DR risk stratification and may serve as a supportive auxiliary indicator for identifying T2DM patients at high DR risk, which may facilitate targeted intervention strategies for diabetic microvascular complications.

## 1 Introduction

Diabetic retinopathy (DR) is a common microvascular complication of diabetes and a leading cause of vision loss and blindness among patients with type 2 diabetes mellitus (T2DM) (1). Early-stage DR often presents without obvious clinical manifestations and is therefore prone to under-diagnosis, which partially accounts for its growing prevalence. Furthermore, DR-related visual impairment and blindness substantially impair patients’ quality of life, raise the risk of comorbidities, and reduce life expectancy (1, 2). Data from a national DR-screening program in Singapore have demonstrated that initial DR symptoms tend to appear roughly 8 years after diabetes onset, with progression across disease stages occurring within 1–2 years (3). Accordingly, novel biomarkers are urgently needed to enhance the performance of early-DR screening.

Elevated serum uric acid (SUA) increases the risk of monosodium urate crystal formation. Accumulating evidence indicates that monosodium urate crystals can activate the NLR family pyrin domain-containing 3 (NLRP3) inflammasome and induce impairment of endothelial nitric oxide-dependent vasodilation (4). The NLRP3 inflammasome releases its canonical products interleukin-1β (IL-1β) and interleukin-18 (IL-18), whereas tumor necrosis factor-α (TNF-α), interleukin-6 (IL-6), and C-reactive protein (CRP) represent broader downstream inflammatory responses.Inflammatory responses play a critical role in the pathogenesis of DR, and elevated SUA exacerbates retinal inflammation and accelerates DR progression (5). Three-quarters of circulating SUA derives from hepatic metabolism of endogenous purines, whereas one-quarter comes from dietary purine intake. Approximately two-thirds of SUA is eliminated via renal excretion; consequently, SUA concentrations are strongly correlated with renal function (6, 7). This important confounder has been overlooked in most prior investigations, highlighting the necessity of adjusting for renal function when evaluating the relationship between SUA and DR. In the present study, we adopted the SUA-to-creatinine ratio (SUA/SCr). This indicator compensates for the influence of renal dysfunction and reflects net endogenous uric-acid production (8), enabling a more accurate assessment of how endogenous urate burden contributes to DR.

This study aimed to investigate the association between SUA/SCr and DR in patients with T2DM. Our findings may support early-DR screening, risk stratification, optimized prognostication, and individualized clinical management for this patient population.

## 2 Methods

### 2.1 Study population

A total of 1091 patients with T2DM admitted to the Department of Internal Medicine at Hefei First People’s Hospital between 2022 and 2024 were initially recruited. After screening based on predefined inclusion and exclusion criteria, 427 patients were finally included in the analysis.

Inclusion criteria:

1. All enrolled patients met the diagnostic criteria for type 2 diabetes mellitus (T2DM) according to the Guideline for the Prevention and Treatment of Type 2 Diabetes Mellitus in China (2020 edition). T2DM was confirmed based on clinical history, fasting plasma glucose, 2-h postprandial glucose, and HbA1c measurements. Patients with a prior established diagnosis of T2DM receiving hypoglycemic treatment were also included. Autoimmune diabetes was excluded by detecting GAD-Ab, IA-2Ab, and insulin autoantibodies. Blood samples for autoantibody detection were collected at baseline before insulin initiation. We acknowledged that prior exogenous insulin administration may interfere with insulin autoantibody results; patients with positive autoantibodies were excluded to rule out autoimmune diabetes.
2. Diabetic retinopathy was diagnosed and graded by dilated fundus examination and fundus photography, following the Early Treatment Diabetic Retinopathy Study Severity Scale.optical coherence tomography (OCT) was only used to evaluate macular structural changes rather than for DR diagnosis or grading. Two masked ophthalmologists independently reviewed the fundus images. Discrepancies were resolved by consensus adjudication.
3. Documented diabetes duration of at least 5 years.

Exclusion criteria:

1. Incomplete clinical data;
2. Other types of diabetes mellitus;
3. Acute diabetic complications or hypoglycemic coma;
4. Ocular diseases unrelated to diabetic retinopathy (DR),including glaucoma, age-related macular degeneration, retinal vein occlusion, and uveitis;
5. Concomitant systemic or immune-mediated disorders;
6. Malignancies, active infection, or severe organ dysfunction (defined as estimated glomerular filtration rate <30 mL/min/1.73 m², severe hepatic dysfunction, or heart failure [NYHA class III–IV]).
7. use of drugs that affect SUA or inflammatory markers, including allopurinol, febuxostat, uricosuric agents, glucocorticoids, and immunosuppressants.

All enrolled patients exhibited insidious-onset diabetes with low tendency toward spontaneous ketosis and a disease duration longer than 5 years. The diagnosis of T2DM was further verified by insulin autoantibody testing together with clinical history. For DR diagnosis, the fundus images were obtained, and all images were interpreted on-site by experienced ophthalmologists from Hefei First People’s Hospital (**Figure 1**).

**Figure 1:**
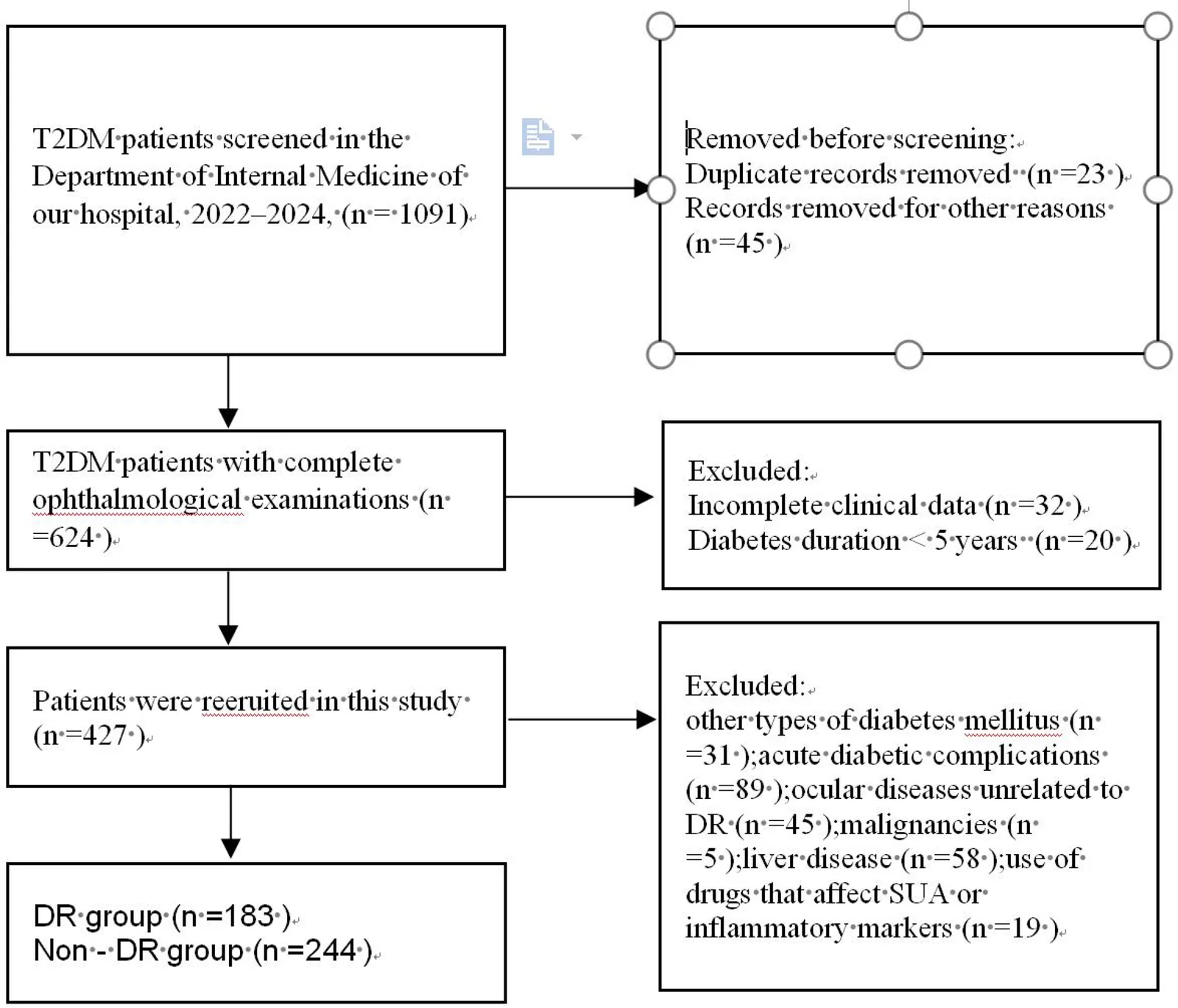
The flow chart of the study population.

### 2.2 Survey and measurements

Demographic and baseline clinical data including sex, age, and diabetes duration were collected for all participants. Biochemical laboratory parameters comprised glycated hemoglobin A1c (HbA1c), fasting plasma glucose (FPG), C-peptide (C-P), albumin (ALB), alanine transaminase (ALT), aspartate transaminase (AST), triglycerides (TG), total cholesterol (TC), low-density lipoprotein cholesterol (LDL-C), gamma-glutamyl transferase (γ-GT), total bilirubin (TBIL), glomerular filtration rate (GFR), serum creatinine (SCr), serum uric acid (SUA), and the serum uric acid-to-creatinine ratio (SUA/SCr).

### 2.3 Statistical analysis

Statistical analyses were conducted using SPSS version 27.0. Normally distributed continuous variables were expressed as mean ± standard deviation (SD) and compared by the independent-samples t-test. Non-normally distributed continuous data were reported as median (interquartile range, IQR) and compared using the Mann-Whitney U test. The standard t-test was applied for data with equal variance, whereas Welch’s t-test was adopted for datasets with unequal variance. Categorical variables were compared using the χ² test. Spearman’s rank-order correlation analysis was utilized to evaluate associations between DR status and relevant clinical variables. Univariable binary logistic regression was performed to screen potential risk factors. Variables with *P* < 0.05 in the univariable analysis were entered into the baseline multivariable logistic regression model. *Post-hoc* power calculation was performed for the primary multivariable logistic regression. With an assumed odds ratio of 1.22, α = 0.05, and a case-to-noncase ratio of 1:1.3, the sample size of 427 participants provided adequate statistical power to detect the target effect size. Receiver-operating-characteristic (ROC) curve analysis was carried out to assess the predictive performance of SUA/SCr for DR. C-statistics were computed and compared via the DeLong test to examine whether adding SUA/SCr could improve the predictive capacity of the baseline DR-risk prediction model. Continuous Net reclassification improvement (NRI) and integrated discrimination improvement (IDI) were further calculated to quantify the incremental predictive value contributed by SUA/SCr. In addition, NRI and IDI analyses are sensitive to sample size, which may limit the reliability of the incremental discriminative performance estimates. A two-tailed *P*-value < 0.05 was considered statistically significant.

## 3 Results

### 3.1 Comparison of baseline characteristics and biochemical indicators

The 427 enrolled patients with T2DM were stratified into two groups based on the presence or absence of DR: 183 participants in the DR group and 244 in the non-DR group. As summarized in **Table 1**, significant between-group differences were detected for age, diabetes duration, HbA1c, ALB, AST, ALT, γ-GT, TBIL, TG, SUA, and SUA/SCr (all *P<*0.05). By contrast, no inter-group differences were noted regarding sex, C-P, FPG, TC, LDL-C, GFR, and SCr (all *P>* 0.05).

**Table 1:** Clinical characteristics of T2DM patients.

Baseline characteristics	DR group	non-DR group	χ² value/t value/U value	P value
Sex/[male/female(%)]	183(48.6)	244(56.6)	2.637	0.104
Age/(years)	60.14 ± 10.54	56.70 ± 10.62	3.32	0.001
Disease duration (years)	14.20 ± 6.73	11.63 ± 5.66	4.01	<0.001
HbA1C/(%)	9.10 ± 2.15	8.54 ± 1.94	2.67	0.008
C-P/(nmol·L−1)	0.57 ± 0.60	0.65 ± 0.41	-1.56	0.119
FPG/(mmol·L^−1^)	8.47 ± 3.77	8.16 ± 2.71	0.92	0.358
ALB/(g·L^−1^)	41.09 ± 3.97	42.73 ± 3.53	-4.41	<0.001
AST/(U·L^−1^)	18.00(15.00, 21.00)	20.00(17.00, 24.75)	0.35	<0.010
ALT/(U·L^−1^)	16.00(12.00, 22.00)	17.00(14.00, 26.75)	0.78	<0.010
γ-GT/(U·L^−1^)	18.00(13.00, 26.00)	22.00(16.00, 33.00)	0.67	<0.010
TBIL/(umol·L^−1^)	15.35 ± 6.96	18.35 ± 6.02	-4.74	<0.001
TG/(mmol·L^−1^)	1.33(0.92, 1.88)	1.46(0.94, 2.24)	0.341	<0.010
TC/(mmol·L^−1^)	4.54 ± 1.30	4.53 ± 1.48	0.06	0.949
LDL-C/(mmol·L^−1^)	2.69 ± 0.97	2.67 ± 0.98	0.20	0.845
GFR/[ml/(min·1.73m²)]	109.01 ± 35.72	106.72 ± 32.47	0.69	0.490
SUA/(μmol·L−1)	295.93 ± 76.31	280.34 ± 74.61	2.11	0.035
SCr/(μmol·L−1)	63.60 ± 15.33	64.74 ± 14.74	-0.78	0.438
SUA/SCr	4.76 ± 1.38	4.44 ± 1.12	2.63	0.009

HbA1c, Glycated hemoglobin; C-P, C-peptide; FPG, Fasting plasma glucose; ALB, Albumin; AST, Aspartate aminotransferase; ALT, Alanine aminotransferase; γ-GT, Gamma-glutamyl transferase; TBIL, Total bilirubin; TG, Triglycerides; TC, Total cholesterol; LDL-C, Low-density lipoprotein cholesterol; GFR, Glomerular filtration rate; SUA, Serum uric acid; SCr, Serum creatinine; SUA/SCr, Serum uric acid-to-creatinine ratio.

### 3.2 Spearman’s correlation analysis between DR and influencing factors

As presented in **Table 2**, among patients with T2DM, DR status showed positive correlations with age, diabetes duration, HbA1c, SUA, and SUA/SCr (all *P* < 0.05). Negative correlations were observed between DR status and C-P, ALB, AST, ALT, γ-GT, and TBIL (all *P* < 0.05). No statistically significant correlations were detected between DR status and sex, FPG, TG, TC, LDL-C, GFR, or SCr (all *P* > 0.05).

**Table 2:** Spearman correlation analysis of influencing factors for DR in patients with T2DM.

Category	Rs	P value
Sex/[male/female(%)]	-0.079	0.105
Age/(years)	0.161	<0.001
Disease duration (years)	0.202	<0.001
HbA1C/(%)	0.132	0.008
C-P/(nmol·L−1)	-0.127	0.013
FPG/(mmol·L^−1^)	0.012	0.813
ALB/(g·L^−1^)	-0.207	<0.001
AST/(U·L^−1^)	-0.208	<0.001
ALT/(U·L^−1^)	-0.150	0.002
γ-GT/(U·L^−1^)	-0.209	<0.001
TBIL/(umol·L^−1^)	-0.286	<0.001
TG/(mmol·L^−1^)	-0.074	0.143
TC/(mmol·L^−1^)	0.026	0.599
LDL-C/(mmol·L^−1^)	0.019	0.708
GFR/[ml/(min·1.73m²)]	0.017	0.724
SUA/(μmol·L−1)	0.106	0.028
SCr/(μmol·L−1)	-0.036	0.458
SUA/SCr	0.101	0.036

HbA1c, Glycated hemoglobin; C-P, C-peptide; FPG, Fasting plasma glucose; ALB, Albumin; AST, Aspartate aminotransferase; ALT, Alanine aminotransferase; γ-GT, Gamma-glutamyl transferase; TBIL, Total bilirubin; TG, Triglycerides; TC, Total cholesterol; LDL-C, Low-density lipoprotein cholesterol; GFR, Glomerular filtration rate; SUA, Serum uric acid; SCr, Serum creatinine; SUA/SCr, Serum uric acid-to-creatinine ratio.

### 3.3 Univariate and multivariable binary logistic regression analysis

Univariate logistic regression analysis was conducted with DR status set as the dependent variable. SUA/SCr was found to be associated with DR among patients with T2DM (*P* <0.05; **Table 3**). Variables screened by univariable binary logistic regression (AST, TBIL, TG, TC, LDL-C, SUA/SCr) were incorporated into the multivariable binary logistic regression analysis. The results demonstrated that TBIL, TG, TC, LDL-C and SUA/SCr were independent influencing factors for DR in patients with T2DM (*P* <0.05; **Table 4**).

**Table 3:** Univariate logistic regression analysis of DR occurrence in patients with T2DM.

Category	B	SE	Wald	Sig	OR	95% CI
Sex/[male/female(%)]	-0.296	0.320	0.851	0.356	0.744	0.397~1.394
Age/(years)	0.019	0.014	1.763	0.184	1.019	0.991~1.048
Disease duration (years)	0.029	0.022	1.654	0.198	1.029	0.985~1.076
HbA1C/(%)	0.077	0.074	1.059	0.303	1.08	0.933~1.249
C-P/(nmol·L−1)	-0.113	0.270	0.174	0.677	0.893	0.526~1.517
FPG/(mmol·L^−1^)	-0.003	0.054	0.003	0.960	0.997	0.897~1.108
ALB/(g·L^−1^)	-0.059	0.039	2.332	0.127	0.943	0.874~1.017
AST/(U·L^−1^)	-0.112	0.037	9.005	0.003	0.894	0.83~0.962
ALT/(U·L^−1^)	0.035	0.020	3.086	0.079	1.035	0.996~1.076
γ-GT/(U·L^−1^)	-0.009	0.009	1.018	0.313	0.991	0.975~1.008
TBIL/(umol·L^−1^)	-0.063	0.024	6.732	0.009	0.939	0.896~0.985
TG/(mmol·L^−1^)	-0.334	0.119	7.932	0.005	0.716	0.568~0.903
TC/(mmol·L^−1^)	1.103	0.378	8.504	0.004	3.014	1.436~6.325
LDL-C/(mmol·L^−1^)	-1.077	0.412	6.836	0.009	0.341	0.152~0.764
GFR/[ml/(min·1.73m²)]	-0.002	0.005	0.154	0.695	0.998	0.988~1.008
SUA/SCr	0.287	0.118	5.884	0.015	1.332	1.057~1.679

HbA1c, Glycated hemoglobin; C-P, C-peptide; FPG, Fasting plasma glucose; ALB, Albumin; AST, Aspartate aminotransferase; ALT, Alanine aminotransferase; γ-GT, Gamma-glutamyl transferase; TBIL, Total bilirubin; TG, Triglycerides; TC, Total cholesterol; LDL-C, Low-density lipoprotein cholesterol; GFR, Glomerular filtration rate; SUA, Serum uric acid; SCr, Serum creatinine; SUA/SCr, Serum uric acid-to-creatinine ratio.

**Table 4:** Multivariable logistic regression analysis of DR occurrence in patients with T2DM.

Category	B	SE	Wald	Sig	OR	95% CI
AST/(U·L^−1^)	-0.027	0.014	3.549	0.06	0.973	0.946~1.001
TBIL/(umol·L^−1^)	-0.09	0.022	17.226	<0.001	0.914	0.876~0.954
TG/(mmol·L^−1^)	-0.321	0.097	10.984	0.001	0.725	0.600~0.877
TC/(mmol·L^−1^)	0.920	0.294	9.790	0.002	2.509	1.410~4.465
LDL-C/(mmol·L^−1^)	-0.909	0.325	7.819	0.005	0.403	0.213~0.762
SUA/SCr	0.198	0.094	4.499	0.034	1.220	1.015~1.465

AST, Aspartate aminotransferase; TBIL, Total bilirubin; TG, Triglycerides; TC, Total cholesterol; LDL-C, Low-density lipoprotein cholesterol; SUA/SCr, Serum uric acid-to-creatinine ratio.

### 3.4 Predictive performance of SUA/SCr for DR in patients with T2DM

Receiver-operating-characteristic (ROC) curve analysis was performed to evaluate the predictive performance of SUA/SCr for DR in patients with T2DM. The corresponding AUC was 0.774 (95% CI: 0.7204–0.8269, *P* <0.05), and the optimal cut-off value was 3.68, with a sensitivity of 79.8% and specificity of 67.2% (**Figure 2A**). SUA/SCr showed statistically significant predictive capacity for DR (*P* <0.001).

**Figure 2:**
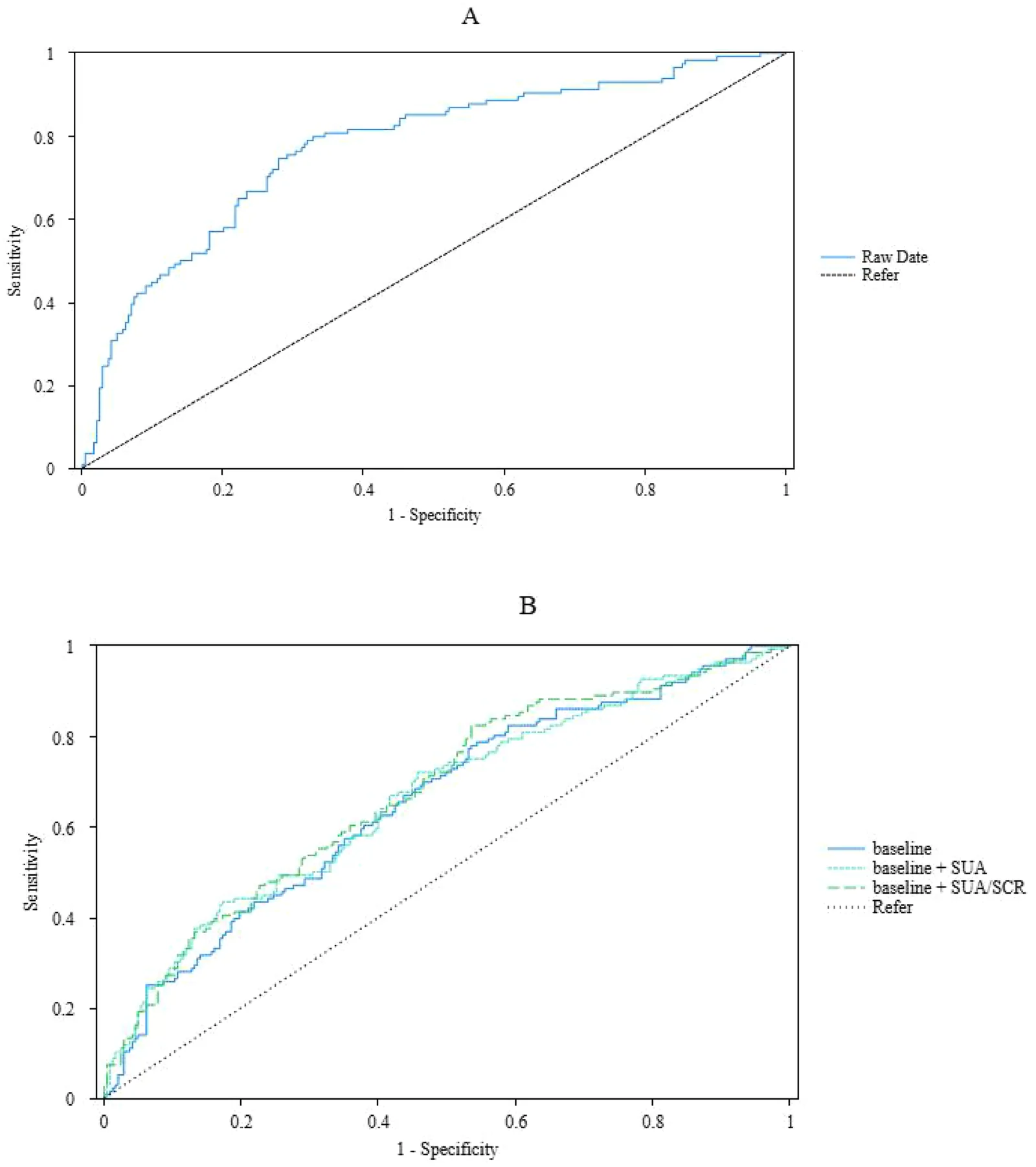
The predictive performance of SUA/SCR for the development of DR in patients with T2DM. Receiver operating characteristic curve analysis of the SUA/SCR for predicting the progression of DR **(A)** and comparison of the C-statistics between different models **(B)**. The baseline risk model includes age, sex, duration of diabetes, and glycated hemoglobin. SUA/SCR, serum uric acid-to-creatinine ratio; DR, diabetic retinopathy; AUC, area under the curve; NRI, net reclassification improvement; IDI, integrated discrimination improvement.

For the baseline model adjusted for sex, age, diabetes duration, and HbA1c, the C-statistic reached 0.655 (95% CI: 0.598–0.713, *P* <0.001). Adding SUA to this baseline model yielded a C-statistic of 0.664 (95% CI: 0.607–0.721, *P* < 0.001), an NRI of 0.253 (95% CI: 0.045–0.462, *P* = 0.017), and an IDI of 0.017 (95% CI: 0.004–0.029, *P* = 0.008). When SUA/SCr was further incorporated into the baseline model, the C-statistic increased to 0.676 (95% CI: 0.620–0.732, *P* < 0.001); the NRI was 0.140 (95% CI: -0.068–0.348, *P* = 0.187), whereas the IDI was 0.021 (95% CI: 0.006–0.036, *P* = 0.006) (**Figure 2B**).

## 4 Discussion

Diabetic retinopathy (DR) represents a major microvascular complication of type 2 diabetes mellitus (T2DM), affecting approximately 30% of patients with diabetes (9). Macular thickening and macular edema are among the leading causes of blindness in patients with DR (10, 11). Early-stage DR frequently remains asymptomatic, which contributes to frequent underdiagnosis in T2DM patients (12). Insufficient health literacy among many patients with T2DM means that macular injury and visual impairment are often detected only at clinical presentation (13). Therefore, early screening for high-risk populations and timely therapeutic interventions are critical for the prevention of DR-related visual loss (14).

Although DR pathogenesis is modulated by multiple risk factors including sex, age, diabetes duration, and glycemic control (15), the relationship between serum uric acid (SUA) and DR remains controversial across existing studies (16). One Mexican cross-sectional study dichotomized SUA levels in T2DM patients and reported that DR prevalence was more than two-fold higher among participants with elevated SUA (≥ 464.0 μmol·L^−1^), compared with those in the normal SUA group (< 464.0 μmol·L^−1^) (16). By contrast, a national cross-sectional investigation involving 2809 participants failed to identify SUA as a risk factor for DR (17). Since circulating SUA concentrations are strongly influenced by renal function, raw SUA measurements cannot faithfully reflect the actual systemic urate burden. Therefore, the present study adopted serum uric acid-to-creatinine ratio (SUA/SCr) as a corrected biomarker to mitigate such renal-related confounding.

Renal function strongly affects SUA levels. It has been proposed that SUA/SCr can reduce the confounding effect of renal function and renal function, and better reflect net urate production after renal adjustment (18). Several studies have demonstrated that SUA/SCr is linked to multiple chronic complications, including fatty liver disease (19), metabolic syndrome (20), renal disease and cardiovascular disease (21). Our study confirmed an association between SUA/SCr and DR presence.Among patients with T2DM, SUA/SCr levels were significantly higher in the DR group than in the non-DR group.This finding is consistent with a 2-year national retrospective study. That study reported an association between elevated SUA and DR among patients with T2DM (22). A cross-sectional study demonstrated that elevated SUA was associated with greater DR risk among T2DM patients. The association was stronger in female patients than in males (5). This discrepancy may be attributed to differences in study populations and unadjusted SUA values.Our study adopted SUA/SCr as a novel marker. Among T2DM patients, SUA/SCr showed a significant positive correlation with DR development across all genders.Furthermore, a Chinese study identified elevated SUA as an independent risk factor for DR in patients with T2DM. Compared with patients with normal SUA, those with SUA above 354.0 μmol/L and 361.0 μmol/L had 1.571-fold and 1.395-fold higher risks of DR, respectively (23). After adjustment for confounders, multivariable logistic regression revealed that elevated SUA/SCr was associated with a 1.220-fold higher odds of DR.Notably, several lipid-related variables exhibited inconsistent statistical significance across unadjusted group comparisons, bivariate Spearman correlation analyses, and multivariable logistic regression models. Such discrepancies are statistically plausible, as unadjusted analyses only evaluate crude associations without controlling for confounding factors, while multivariable regression quantifies the independent relationship between each variable and DR after adjusting for relevant clinical covariates.

Existing evidence indicates that SUA/SCr possesses predictive value for nonalcoholic Fatty Liver Disease (NAFLD). It can serve as a marker for risk stratification and early intervention in NAFLD patients (24). Furthermore, studies have demonstrated that SUA/SCr enhanced the discriminative performance of cardiovascular mortality models among hemodialysis patients (25). ROC curve analysis yielded an AUC of 0.774 for SUA/SCr in discriminating DR. This result suggested that SUA/SCr had moderate predictive efficacy for DR.We established a baseline model with the C-statistic used to quantify model discrimination. This model incorporated sex, age, diabetes duration and HbA1c.After SUA was incorporated into the model, the C-statistic was 0.664 (NRI = 0.253, *P* = 0.017; IDI = 0.017, *P* = 0.008). These results indicated that SUA improved DR risk prediction for T2DM patients.After adding SUA/SCr to the model, although the IDI reached statistical significance, the NRI did not. Such results suggest that SUA/SCr may provide limited supplementary predictive information beyond SUA, rather than substantially enhancing model discrimination. Further large-scale studies are required to validate its predictive utility.

. SUA exhibits dual redox properties and may exert context-dependent antioxidant or pro-oxidant biological effects; therefore, elevated SUA does not directly trigger oxidative stress. Clinical evidence regarding the association between SUA and systemic oxidative stress remains inconsistent. Kaya et al. found no significant differences in ischemia-modified albumin, total oxidant capacity, or total antioxidant capacity between uncomplicated T2DM patients with elevated SUA and those with normal SUA (26). The relationship between SUA and oxidant-antioxidant balance may depend on the clinical context and the presence of diabetic vascular complications. In patients with DR, elevated SUA may shift toward pro-oxidant activity, promoting retinal inflammatory injury and participating in the pathological process of DR (27). Hyperglycemia triggers oxidative stress, inflammation, accumulation of advanced glycation end products (AGEs) and upregulation of vascular endothelial growth factor (VEGF). These are core drivers underlying the pathogenesis of DR (21, 28–31). Elevated SUA is closely linked to diabetic microvascular complications, including DR, diabetic nephropathy and diabetic peripheral neuropathy.Elevated SUA suppresses endothelial nitric oxide synthesis and activates the renin-angiotensin system (5). Furthermore, under hyperglycemic conditions, it may exacerbate retinal inflammation and activate the Notch signaling pathway, potentially contributing to DR presence (32).

This study has several limitations. First, we cannot establish a causal or directional relationship between SUA/SCr and DR. Reverse causality may exist: renal and vascular alterations accompanying DR could affect renal urate excretion and urate handling, which in turn influence serum uric acid levels and the SUA/SCr ratio.As a single-center cross-sectional investigation, it can only demonstrate associations rather than causality between SUA/SCr and DR. The limited sample size and single regional enrollment may introduce selection bias. Furthermore, uncollected information regarding hypoglycemic agents, diet and physical activity may cause residual confounding. Further large-sample, high-quality prospective and preclinical studies across diverse populations and regions are required to validate our findings and generate more robust evidence.

In conclusion, SUA/SCr was identified as an independent risk factor for DR among patients with T2DM. SUA/SCr provides limited supplementary discriminative information for identifying individuals at higher DR risk and may serve as an auxiliary indicator for DR risk stratification. When integrated with comprehensive ophthalmic evaluations, including routine visual acuity testing, dilated fundus examination, fundus photography, and OCT assessment, SUA/SCr may help optimize individualized patient management. Given the cross-sectional, single-center design and the modest, inconsistent incremental discriminative performance of SUA/SCr in the present study, further large-scale, prospective, and externally validated studies are essential to confirm its clinical utility.

## Data Availability

The raw data supporting the conclusions of this article will be made available by the authors, without undue reservation.

## References

[1] Hou X Wang L Zhu D Guo L Weng J Zhang M . Prevalence of diabetic retinopathy and vision-threatening diabetic retinopathy in adults with diabetes in China. Nat Commun. (2023) 14:4296. doi: 10.1038/s41467-023-39864-w 37463878 PMC10354077

[2] Li S Ye E Huang J Wang J Zhao Y Niu D . Global, regional, and national years lived with disability due to blindness and vision loss from 1990 to 2019: Findings from the Global Burden of Disease Study 2019. Front Public Health. (2022) 10:1033495. doi: 10.3389/fpubh.2022.1033495 36388337 PMC9650182

[3] Jonas JB . Diabetic retinopathy. Asia Pac J Ophthalmol (Phila). (2024) 13:100077. doi: 10.1016/j.apjo.2024.100077 38795867

[4] Li X Huang B Liu Y Wang M Cui JQ . Uric acid in diabetic microvascular complications: Mechanisms and therapy. J Diabetes Complications. (2025) 39:108929. doi: 10.1016/j.jdiacomp.2024.108929 39689504

[5] Hu Y Chan Z Li C Shi Y She X Gu C . Higher serum uric acid levels are associated with an increased risk of vision-threatening diabetic retinopathy in type 2 diabetes patients. Invest Ophthalmol Vis Sci. (2021) 62:23. doi: 10.1167/iovs.62.4.23 33861320 PMC8083112

[6] Li Z Su Y Su H Pan J Li S Lu L . Serum uric acid and its metabolism-a vital factor in the inflammatory transformation of cancer. J Adv Res. (2026) 83:869–96. doi: 10.1016/j.jare.2025.08.031 40850676 PMC13131520

[7] Yanai H Adachi H Hakoshima M Katsuyama H . Molecular biological and clinical understanding of the pathophysiology and treatments of hyperuricemia and its association with metabolic syndrome, cardiovascular diseases and chronic kidney disease. Int J Mol Sci. (2021) 22:9221. doi: 10.3390/ijms22179221 34502127 PMC8431537

[8] Zhou X Wang Z Zhu Y Feng S Wu H Zhu D . Effect of serum uric acid to creatinine ratio on cognitive function decline in middle-aged adults: Longitudinal evidence from CHARLS. J Alzheimers Dis. (2025) 103:582–92. doi: 10.1177/13872877241303789 39791192

[9] Zhou J Chen B . Retinal cell damage in diabetic retinopathy. Cells. (2023) 12:1342. doi: 10.3390/cells12091342 37174742 PMC10177610

[10] Prince J Kumar D Ghosh A Arevalo JF Zhang AY . Surgical management of diabetic macular edema. Curr Diabetes Rep. (2023) 23:119–25. doi: 10.1007/s11892-023-01505-3 37043090

[11] Tatsumi T . Current treatments for diabetic macular edema. Int J Mol Sci. (2023) 24:9591. doi: 10.3390/ijms24119591 37298544 PMC10253534

[12] Bryl A Mrugacz M Falkowski M Zorena K . The effect of diet and lifestyle on the course of diabetic retinopathy-a review of the literature. Nutrients. (2022) 14:1252. doi: 10.3390/nu14061252 35334909 PMC8955064

[13] Thapa R Sharma S Pradhan E Duwal S Poudel M Shrestha KG . Prevalence and associated factors of diabetic retinopathy among people with diabetes screened using fundus photography at a community diabetic retinopathy screening program in Nepal. BMC Ophthalmol. (2023) 23:429. doi: 10.1186/s12886-023-03173-z 37872518 PMC10594880

[14] Zureik A Julla JB Erginay A Vidal-Trecan T Juddoo V Gautier JF . Prevalence, severity stages, and risk factors of diabetic retinopathy in 1464 adult patients with type 1 diabetes. Graefes Arch Clin Exp Ophthalmol. (2021) 259:3613–23. doi: 10.1007/s00417-021-05298-7 34264396

[15] Wang W Ji Q Ran X Li C Kuang H Yu X . Prevalence and risk factors of diabetic peripheral neuropathy: A population-based cross-sectional study in China. Diabetes Metab Res Rev. (2023) 39:e3702. doi: 10.1002/dmrr.3702 37490047

[16] Rivera-De-la-Parra D Hernández-Jiménez S Almeda-Valdés P Aguilar-Salinas CA Graue-Hernández EO Pérez-Peralta L . Association between uric acid and referable diabetic retinopathy in patients with type 2 diabetes. Sci Rep. (2024) 14:12968. doi: 10.1038/s41598-024-63340-0 38839883 PMC11153536

[17] Hu Y Li Q Min R Deng Y Xu Y Gao L . The association between serum uric acid and diabetic complications in patients with type 2 diabetes mellitus by gender: a cross-sectional study. PeerJ. (2021) 9:e10691. doi: 10.7717/peerj.10691 33520463 PMC7811288

[18] Wang Q Wang Z Sun Y Tang F Lu B Zhou K . The serum uric acid to serum creatinine ratio is an independent risk factor for type 2 diabetes in community-dwelling elderly: A longitudinal analysis. Nutr Metab Cardiovasc Dis. (2024) 34:2749–56. doi: 10.1016/j.numecd.2024.08.018 39353840

[19] Wang F Zheng X Ni X Li Y . Increased serum uric acid to creatinine ratio is associated with metabolic dysfunction associated steatotic liver disease. Sci Rep. (2025) 15:26941. doi: 10.1038/s41598-025-09073-0 40707569 PMC12290112

[20] Cao T Tong C Halengbieke A Ni X Tang J Zheng D . Serum uric acid to creatinine ratio and metabolic syndrome in middle-aged and elderly population: Based on the 2015 CHARLS. Nutr Metab Cardiovasc Dis. (2023) 33:1339–48. doi: 10.1016/j.numecd.2023.05.004 37248143

[21] Gigante A Assanto E Brigato C Pellicano C Iannazzo F Rosato E . Clinical outcomes in patients with cardiorenal multimorbidity: the role of serum uric acid/serum creatinine ratio. High Blood Press Cardiovasc Prev. (2025) 32:209–16. doi: 10.1007/s40292-025-00706-z 40035942 PMC11890324

[22] Chen D Sun X Zhao X Liu Y . Associations of serum uric acid and urinary albumin with the severity of diabetic retinopathy in individuals with type 2 diabetes. BMC Endocr Disord. (2020) 20:467. doi: 10.1186/s12886-020-01713-5 33256661 PMC7706232

[23] Wang H Gu L Ma Y Xing X Qu Y Shi X . High levels of serum uric acid are associated with microvascular complications in patients with long-term diabetes. Diabetol Metab Syndr. (2025) 17:106. doi: 10.1186/s13098-025-01656-1 40148898 PMC11951721

[24] El-Sehrawy AAMA Alshkarchy SS Kareem AK Maashi MS Al-Khafaji ZA Hjazi A . Association between serum uric acid to creatinine ratio with metabolic profile in subjects with non-alcoholic fatty liver disease (NAFLD). BMC Endocr Disord. (2025) 25:260. doi: 10.1186/s12902-025-02074-0 41225452 PMC12613905

[25] Huang W Ding N Zhang Y Qiu D Wei L Zhao C . Association between the serum uric acid-to-creatinine ratio and cardiovascular mortality in maintenance hemodialysis patients. Ren Fail. (2025) 47:2509787. doi: 10.1080/0886022X.2025.2509787 40415215 PMC12107642

[26] Kaya EK Çandar T Ergün İ . Assessment of the relationship between serum uric acid levels and oxidative stress markers in patients with uncomplicated type 2 diabetes mellitus. Acta Med. (2024) 55:247–54. doi: 10.32552/2024.ActaMedica.1036

[27] Du L Zong Y Li H Wang Q Xie L Yang B . Hyperuricemia and its related diseases: mechanisms and advances in therapy. Signal Transduct Target Ther. (2024) 9:212. doi: 10.1038/s41392-024-01916-y 39191722 PMC11350024

[28] Seo H Park SJ Song M . Diabetic retinopathy (DR): Mechanisms, current therapies, and emerging strategies. Cells. (2025) 14:376. doi: 10.3390/cells14050376 40072104 PMC11898816

[29] Yun JH . Interleukin-1β induces pericyte apoptosis via the NF-κB pathway in diabetic retinopathy. Biochem Biophys Res Commun. (2021) 546:46–53. doi: 10.1016/j.bbrc.2021.01.108 33571904

[30] Qiu AW Wang NY Yin WJ Zhu ZQ Liu QH Zhang WW . Retinal Müller cell-released exosomal MiR-92a-3p delivers interleukin-17A signal by targeting Notch-1 to promote diabetic retinopathy. Invest Ophthalmol Vis Sci. (2025) 66:1. doi: 10.1167/iovs.66.1.1 39745681 PMC11702826

[31] Tanaka K Yamanaga S Hidaka Y Nishida S Kinoshita K Kaba A . Long-term impact of baseline serum uric acid levels on living kidney donors: a retrospective study. BMC Nephrol. (2021) 22:89. doi: 10.1186/s12882-021-02295-0 33711960 PMC7953616

[32] Yang J Liu Z . Mechanistic pathogenesis of endothelial dysfunction in diabetic nephropathy and retinopathy. Front Endocrinol (Lausanne). (2022) 13:816400. doi: 10.3389/fendo.2022.816400 35692405 PMC9174994

